# A non-linear reverse-engineering method for inferring genetic regulatory networks

**DOI:** 10.7717/peerj.9065

**Published:** 2020-04-29

**Authors:** Siyuan Wu, Tiangang Cui, Xinan Zhang, Tianhai Tian

**Affiliations:** 1 School of Mathematics, Monash University, Clayton, VIC, Australia; 2 School of Mathematics and Statistics, Central China Normal University, Wuhan, PR China

**Keywords:** Genetic regulatory network, Network inference, Hematopoiesis, Probabilistic graphic model, Differential equation

## Abstract

Hematopoiesis is a highly complex developmental process that produces various types of blood cells. This process is regulated by different genetic networks that control the proliferation, differentiation, and maturation of hematopoietic stem cells (HSCs). Although substantial progress has been made for understanding hematopoiesis, the detailed regulatory mechanisms for the fate determination of HSCs are still unraveled. In this study, we propose a novel approach to infer the detailed regulatory mechanisms. This work is designed to develop a mathematical framework that is able to realize nonlinear gene expression dynamics accurately. In particular, we intended to investigate the effect of possible protein heterodimers and/or synergistic effect in genetic regulation. This approach includes the Extended Forward Search Algorithm to infer network structure (top-down approach) and a non-linear mathematical model to infer dynamical property (bottom-up approach). Based on the published experimental data, we study two regulatory networks of 11 genes for regulating the erythrocyte differentiation pathway and the neutrophil differentiation pathway. The proposed algorithm is first applied to predict the network topologies among 11 genes and 55 non-linear terms which may be for heterodimers and/or synergistic effect. Then, the unknown model parameters are estimated by fitting simulations to the expression data of two different differentiation pathways. In addition, the edge deletion test is conducted to remove possible insignificant regulations from the inferred networks. Furthermore, the robustness property of the mathematical model is employed as an additional criterion to choose better network reconstruction results. Our simulation results successfully realized experimental data for two different differentiation pathways, which suggests that the proposed approach is an effective method to infer the topological structure and dynamic property of genetic regulations.

## Introduction

Hematopoiesis is a highly complex process that controls the proliferation, differentiation and maturation of hematopoietic stem cells (HSCs) (Ng & Alexander, 2017). It has been widely accepted that genetic regulatory networks control the developmental processes of various types of blood cells (Cedar & Bergman, 2011). Although the regulatory mechanisms have been studied over a century, there are still many challenging questions regarding the cell fate determination in hematopoiesis (Ottersbach et al., 2010). Thus, it is imperative to unravel the regulatory mechanisms for the study of hematopoiesis.

In adult mammals, hematopoiesis occurs mostly in the bone marrow (Birbrair & Frenette, 2016). HSCs have the feature of self-renewal and multipotent as well as the ability to differentiate into multipotent progenitors (MPPs). Then, MPPs will differentiate into two main lineages of blood cells, namely the myeloid lineage which starts at common myeloid progenitors (CMPs) and the lymphoid lineage which starts at common lymphoid progenitors (CLPs). In addition, the myeloid lineage has two distinct progenitors, namely megakaryocyte-erythroid progenitors (MEPs) and granulocyte-macrophage progenitors (GMPs). MEPs can differentiate into megakaryocytes and erythrocytes, and GMPs can give rise to mast cells, macrophages and granulocytes. Lymphoid lineage cells include T lymphocytes (T-cells), B lymphocytes (B-cells) and natural killer cells (NK-cells) (Orkin & Zon, 2008). In this work, we focus on the fate determination of HSCs in the myeloid lineage for the choice between erythrocytes and neutrophils.

During the developmental process, a number of transcriptional factors (TFs) act as regulators to control the fate determination of HSCs (Aggarwal et al., 2012). Among them, the genetic complex Gata1-Gata2-PU.1 is a very important module for the cell-fate choice of CMPs between erythrocytes or neutrophils (Friedman, 2007; Liew et al., 2006; Ling et al., 2004). In particular, the Gata1-PU.1 complex forms a double negative feedback module, in which each gene inhibits the expression of the other (Friedman, 2007). Recently it has been elucidated that the fate determination of HSCs was defined not only by the ratio of Gata1 and PU.1 (Hoppe et al., 2016), but also by a third party during the regulation. For example, *FOG-1* is a significant third party to regulate the Gata1-PU.1 module (Chang et al., 2002; Mancini et al., 2012). Erythropoietin receptor (EpoR) signaling also acts the essential role in regulating the Gata1-PU.1 Module (Zhao et al., 2006). Although the regulatory mechanisms of the Gata1-Gata2-PU.1 complex in hematopoiesis are relatively well studied, the connection of this triad complex with other significant genes as well as the role of these genes in hematopoiesis are mostly unclear (Liew et al., 2006).

Mathematical modeling is an important method for inferring the detailed regulatory mechanisms. In 1910, Archibald Hill proposed a classical non-linear ordinary differential equation (ODE or Hill equation) model to describe the sigmoidal oxygen binding curve of hemoglobin (Hill, 1910). Since then, the Hill equation has been applied to explore the mechanisms in a wide range of genetic regulatory networks and biological systems. For example, the genetic toggle switching was achieved by the models with Hill equations (Gardner, Cantor & Collins, 2000). In addition, the Hill equation was employed to formalize the mechanisms of cell fate determination (Xiong & Ferrell, 2003; Laslo et al., 2006; Huang et al., 2007). Recently, the Hill equation was also used to discover a regulatory network of 52 genes with the uniform activation and repression strengthes (Li & Wang, 2013). Another widely used approach is the Shea–Ackers formalism for studying the thermodynamics of regulatory networks (Shea & Ackers, 1985). We developed a mathematical model based on the Shea–Ackers formalism to study the regulations of the Gata1-Gata2-PU.1 complex (Tian & Smith-Miles, 2014). A stochastic model was also proposed to explore the function of noise in regulating the fate determination of HSCs. Simulations suggested that fluctuations of protein numbers may lead the HSC to different developmental pathways. In recent years substantial process has been made to design various types of mathematical models for describing the regulatory mechanisms of gene networks, including stochastic differential equations, stochastic kinetic systems, qualitative differential equations, Michaelis–Menten formalism, S-system and power-law formalism (de Jong, 2002; Liu & Wang, 2008; Wang & Tian, 2010; Maetschke et al., 2013; Woods et al., 2016; Olariu & Peterson, 2018; Yang et al., 2018; Yang & Bao, 2019). In particular, a number of mathematical models have been designed to realize the stable states of gene expression levels in the differentiation of HSCs (Chang et al., 2006; Huang et al., 2007; Chickarmane, Enver & Peterson, 2009; Olariu & Peterson, 2018). However, the majority of these models only considered the functions of each gene independently, namely variable *x*_*i*_ for the expression level of gene *i* in the model is in the form of \sum\nolimits_i {{a_i}} {x_i}. Nonetheless, this type of models fails to represent the co-operation functions of genes together. There is a lack of investigations for the effect of possible protein heterodimers and/or synergistic effect in genetic regulations, namely variables *x*_*i*_ in the model are also in the form of \sum\nolimits_{i,j} {{b_{ij}}} {x_i}{x_j}. Most recently, single-cell studies have been conducted to explore the hematopoietic system. Compared with the analysis of bulk cells, the advantage of single-cell analysis is the ability to understand the heterogeneity within the cell population (Guo et al., 2010; Ye, Huang & Guo, 2017). With the development of single-cell analysis, researchers have raised more novel computational and statistical methods to explore the regulatory mechanism of hematopoiesis. For example, the partial correlation method, Boolean model and ODE model were employed to construct the genetic regulatory networks from the single-cell expression profiles (Hamey et al., 2017; Wei et al., 2017). In addition, a deep learning method was applied to unravel the fate decision in hematopoiesis (Athanasiadis et al., 2017).

Recently, we proposed a general approach that combines both top-down and bottom-up approaches to reconstruct the genetic regulatory networks of the fate choice between erythrocytes and neutrophils (Wu, Cui & Tian, 2018). The key issue in this work includes a large number of unknown parameters and a high computational cost to add potential regulations. For the issue of parameter number, a linear ODE model may have the least number of unknown parameters among the models for all possible regulations between genes. However, since the linear model is limited to describe the linear relationship, it is not appropriate to use the linear model to study systems with complex non-linear dynamics. Although the non-linearity has been addressed by the reverse-engineering methods with the cost of more unknown parameters (Chickarmane & Peterson, 2008; Crombach et al., 2012; Meister et al., 2013; Li & Wang, 2013; Wang et al., 2016), the issue of protein heterodimers and/or synergistic effect between genes has not been discussed in the majority of literature at all. This work is designed to address these issues by proposing a novel approach for reconstructing genetic regulatory networks. The first innovation of this approach is the new non-linear ODE model as the bottom-up approach to study the effect of protein heterodimers and/or synergistic effect explicitly. The second innovation of this work is the proposed Extended Forward Search Algorithm as the top-down approach to infer the structure of networks in our newly proposed non-linear model. The proposed approach thus is able to not only reduce the complex structure of genetic regulatory networks but also improve the inference efficiency substantially because the number of parameters in the mathematical model is decreased. We examined the capability of our proposed method by studying the genetic regulatory networks for the fate determination of HSCs.

## Methods

### Experimental data

**Figure 1:**
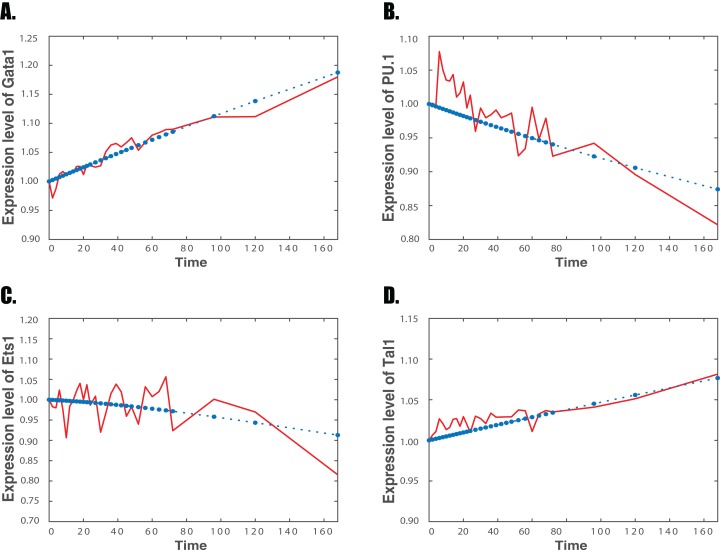
Simulation results and experimental data of the regulatory network for erythrocyte differentiation Red solid line: experimental microarray data; Blue star dash line: simulation of the regulatory network. (A) Gene *Gata1*; (B) gene *PU.1*; (C) gene *Ets1*; (D) gene *Tal1*.

**Figure 2:**
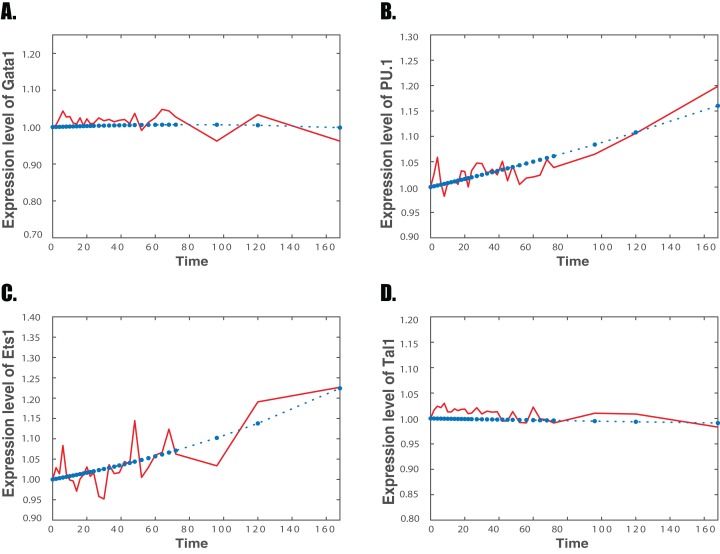
Simulation results and experimental data of the regulatory network for neutrophil differentiation Red solid line: experimental microarray data; Blue star dash line: simulation of the regulatory network. (A) Gene *Gata1*; (B) gene *PU.1*; (C) gene *Ets1*; (D) gene *Tal1*.

In this work, we used the sub-series GSE49987 as the experimental data from the published microarray dataset GSE49991 (May et al., 2013). This dataset contains the expression profiles collected by experiments using the cell line FDCPmix. This dataset was generated with the probe name version of Agilent Whole Mouse Genome Microarray 4 × 44 K (May et al., 2013). It provides microarray gene expression profiles of hematopoietic stem cells (HSCs) differentiating into erythrocytes and neutrophils. This microarray dataset is available at https://www.ncbi.nlm.nih.gov/geo/query/acc.cgi?acc=GSE49991. To convert all microarray probe IDs to gene names, we pre-processed this dataset based on the Ensembl BioMart and GO Enrichment Analysis (The Gene Ontology Consortium, 2017). From a previous study, the regulatory network of 18 core genes during the hematopoiesis has been curated (Moignard et al., 2013). Moreover, the same research team studied the regulatory interaction of 26 core genes during the hematopoiesis (Moignard et al., 2015). The total number of distinct genes in these two studies is 30. Thus, in our work we considered 30 genes whose names are listed in Table S1. There are three repeated experiments for each developmental process, each of which contains the expression levels of 30 genes from HSCs to differentiated cells at 30 time points spanning over 1 week. The observation time points are those starting from the HSCs/progenitors stage (1 point), then every 2 h over the first day (12 points), every 3 h over the second day (8 points), every 4 h over the third day (6 points), every 24 h until the fifth day (2 points), and the seventh day (1 point). In this study, we used the average data of these three repeated tests as the experimental data for each time point. The time points and expression data of four genes can be found in the following Figs. 1 and 2.

### Selection of candidate genes

Based on our research experience (Wang et al., 2016), it is challenging to study a dynamic network with 30 genes. Thus, we conducted an extensive literature review for selecting a smaller number of important genes based on their relationship with the three genes *Gata1, Gata2* and *PU.1*. These candidate genes should be essential for the cell-fate choice in hematopoiesis, or they significantly interact with these three genes. For example, gene *Scl/Tal1* interacts with *Gata1, Eto2/Cbfa2t3* and *Ldb1* (Goardon et al., 2006), and is a regulator in the differentiation of hematopoietic stem cells (HSCs) (Shivdasani, Mayer & Orkin, 1995; Zhang et al., 2005; Porcher et al., 1996; Real et al., 2012). In addition, *Eto2/Cbfa2t3* regulates the differentiation of HSCs by repressing the expression of target gene *Scl/Tal1* (Goardon et al., 2006). Moreover, *Ldb1* is a significant transcriptional factor (TF) for the differentiation of erythroid lineage (Soler et al., 2010). According to the ChIPSeq analysis, *Ldb1* is necessary for HSCs to control their maintenance since it binds to the majority of enhancer elements in hematopoiesis (Li et al., 2011).

We also included a number of genes with potential regulatory relationship with the three genes *Gata1, Gata2* and *PU.1*. For example, it was indicated that there might be unclear regulations between *Gata2* and *Gfi1* (Moignard et al., 2013). *Gfi1* is an important TF in the regulation of HSCs differentiation (van der Meer, Jansen & van der Reijden, 2010; Lancrin et al., 2012). *Gfi1* is required for the differentiation of common lymphoid progenitors (CLPs) and common myeloid progenitors (CMPs) from HSCs and exists in the majority of HSCs, CLPs and CMPs. Similar to gene *Gfi1*, gene *Runx1* is also expressed in most HSCs and progenitor cells as well. Then, *Gfi1* and/or *Runx1* are expressed continually in most cells which differentiate into the granulocyte lineage (North et al., 2004). *Lmo2* is a master regulator of hematopoiesis (Inouea et al., 2013). However, its specific role in regulation is still unclear. Experimental studies suggested that the knockdown of *Lmo2* does not affect the expression of *Gata1* and *Scl/Tal1* (Inouea et al., 2013). However, the overexpression of *Lmo2* gene also inhibited erythroid differentiation (Visvader et al., 1997). In addition, gene *Ets1* is a suppressor in the erythrocyte differentiation. It is downregulated in erythrocyte differentiation by binding to and activating the Gata2 promoter (Lulli et al., 2006). The last candidate gene is *Notch1* that inhibits the differentiation of granulocyte lineage by maintaining the expression of gene *Gata2*. It also enhances the HSCs differentiate to CLPs (Kumano et al., 2001; Stier et al., 2002). Therefore, in this study we considered the regulatory networks with the following 11 genes: *Gata1, Gata2, PU.1/Sfpi1, Runx1, Eto2/Cbfa2t3, Ets1, Notch1, Scl/Tal1, Ldb1, Gfi1* and *Lmo2*. The detailed information of the references for these 11 genes is also given in Table S2 in Supplemental Information.

### Top-down approach: extended forward search algorithm

To reduce the number of unknown parameters in our proposed mathematical model, we used the probabilistic graphical models as the top-down approach to infer the topological structure of gene regulatory networks. Probabilistic graphical model is a useful tool for inferring the network structure (Noor et al., 2013). One type of probabilistic graphical models is the Gaussian graphical model (GGM), which provides a simple and effective method to characterize the regulatory relationship between genes. The GGM is based on the calculation of the conditional dependencies among genes using the gene expression data. The edge connecting two genes in the model is neglected if they are conditionally independent given all other genes (Krämer, Schäfer & Boulesteix, 2009). In this work it is assumed that a system includes genes {*G*_1_, …, *G*_*m*_} with expression levels *x*_*ij*_ for gene *G*_*i*_ at time point *j*. Compared with the existing methods that study networks with genes only, this work will study gene networks that include not only genes in the form of monomers {*G*_1_, …, *G*_*m*_}, which are represented by the linear terms in the model, but also protein heterodimers and/or synergistic effect {*G*_*k*_*–G*_*l*_} (*k*, *l* = 1,…,*m*), which are represented by the non-linear terms (NLTs) in the model. There are two reasons for using the NLTs {*G*_*k*_*–G*_*l*_}. Firstly, we can use the product of two variables to represent the synergistic effect of these two genes. Secondly, if the NLT represents the protein heterodimer, we assumed that the binding and disassociation reactions for the heterodimer {*G*_*k*_*–G*_*l*_} reach an equilibrium state quickly. Thus the level of the heterodimer {*G*_*k*_*–G*_*l*_} can be written as *C*_*kl*_ × *G*_*k*_ × *G*_*l*_, where *C*_*kl*_ is the equilibrium constant. We can consider this constant *C*_*kl*_ as a coefficient in our mathematical model. In both cases, we only need to consider the product of the expression levels of these two genes, namely *y*_*klj*_ = *x*_*kj*_*x*_*lj*_, as the level of NLT {*G*_*k*_*–G*_*l*_} at time *t*_*j*_ for our algorithm computation. Since the number of possible regulations from NLTs to genes is much larger than that of possible regulations among genes (i.e. 726 vs 110), the regulations from NLTs to genes will dominate the whole genetic regulatory system with high probability. However, the regulations among genes should be the core mechanisms rather than the regulations from NLTs to genes. To avoid the dominance of NLTs regulations, we assume that the number of regulations from NLTs to genes does not exceed that between genes.

According to the GGM (Wang, Myklebost & Hovig, 2003; Wang et al., 2016), we proposed a new algorithm, named Extended Forward Search Algorithm (EFSA), to infer the topological structure of regulatory networks that includes both genes and NLTs. Let **X** = (*x*_1_, *x*_2_, …, *x*_N_) be a vector that consists of *m* genes and *n* NLTs (*N* = *m* + *n*). The following three matrices are constructed, namely a *m* × *m* covariance matrix **A** of *m* genes, a *m* × *n* covariance matrix **B** to measure the covariance between *m* genes and *n* NLTs, and a *n* × *n* covariance matrix **C** of *n* NLTs. The *N*-dimensional matrix **M** is defined by

(1) $${\bf M} = {\rm }\left[ {\matrix{ {\bf A} & {\bf B} \cr {{\bf B}^{\prime}} & {\bf C} \cr } } \right],$$

where **B**′ is the transpose of **B**. An initial empty graph **G** is built by the *N*-dimensional identity matrix. This initial graph **G** consists of four matrices **G**_1_, **G**_2_, **G**_3_ and **G**_4_ which have the same dimensions as **A**, **B**, **B**′ and **C**, respectively, namely

(2) $${\bf G} = {\rm }\left[ {\matrix{ {{{\bf G}_1}} & {{{\bf G}_2}} \cr {{{\bf G}_3}} & {{{\bf G}_4}} \cr } } \right],$$

where **G**_1_ and **G**_4_ are identity matrix with dimensions *m* and *n*, respectively, and **G**_2_ and **G**_3_ are *m* × *n* and *n* × *m* zero matrices, respectively.

The proposed algorithm is given below.

### Algorithm 1: extended forward search algorithm

1. Let **X** = (*x*_1_,*x*_2_,…, *x*_*N*_) be a vector with *N* elements, and *N* be the number of components consist of *m* genes and *n* NLTs. An initial empty graph **G** is built by the *N*-dimensional identity matrix, which is defined by Eq. (2).
2. Substitute all covariance values from the diagonal positions of sub-matrix **A** into the corresponding positions of sub-matrix **G**_1_, and then based on the updated **G**_1_, use the Iterative Maximum Likelihood Estimates Algorithm (IMLEA) to compute the new covariance matrix (Dempster, Laird & Rubin, 1977).
3. Add an undirected edge $E_{ij}^1$ ((*i*, *j*) ∈ [1, *m*]^2^) into **G**_1_, namely add the symmetrical covariance value between the *i*th gene and *j*th gene from the positions **A**(*i*, *j*) and **A**(*j*, *i*) into the positions **G**_1_(*i*, *j*) and **G**_1_(*j*, *i*), respectively. Then compute a new covariance matrix by the IMLEA. Based on the deviance difference between the new covariance matrix and that before addition, test the significance of the added edge $E_{ij}^1$ by using the Chi-square distribution with one degree of freedom. The *p*-value of the Chi-square test is used in the next step as the edge selection criterion. Record the *p*-value of this tested edge and remove it from **G**_1_.
4. Add a new undirected edge into **G**_1_. Then, repeat the computation in Step 3. After all possible undirected edges have been tested, sort all tested edges in ascending order by their *p*-values. If the smallest *p*-value is lower than the predefined cut-off value, add the edge with the smallest *p*-value into the sub-graph **G**_1_ permanently.
5. Go back to step 3, add the second edge in the updated sub-graph **G**_1_. Repeat the computation in steps 3 and 4 until the smallest *p*-value of an added edge is larger than the cutoff *p*-value.
6. Based on the last updated undirected graph **G**_1_, the graph orientation rules are applied to transform the undirected graph into a directed acyclic graph (DAG) (Meek, 1995). The inferred DAG with *m*_1_ directed edges, denoted as **A**_**s**_, represents the predicted regulatory network among *m* genes.
7. Test the possible edges between *m* genes and *n* NLTs. Based on the latest matrix **G**, add an undirected edge $E_{ij}^2$ between the *i*th gene and the *j*th NLT. That is, add the symmetrical covariance value between the *i*th gene and *j*th NLT from the positions **B**(*i*, *j*) and **B**′(*j*, *i*) into the positions **G**_2_(*i*, *j*) and **G**_3_(*j*, *i*), respectively. Then, compute a new covariance matrix by the IMLEA. Based on the deviance difference between the new covariance matrix and that before addition, test the significance of the added edge $E_{ij}^2$ by using the Chi-square distribution with one degree of freedom. The *p*-value of the Chi-square test is used as the edge selection criterion. Record the *p*-value of this tested edge $E_{ij}^2$ and remove it from **G**.
8. Repeat the computation in steps 7 for the regulation between genes and NLTs. The last updated sub-graph **G**_3_ with *n*_1_ edges, denoted as **B**′_**s**_, is the predicted directed regulatory network from *n* NLTs to *m* genes. Since we only consider regulations among genes and those from NLTs to genes, the result matrix is given as follows:
  (3) $${{\bf G}_{\bf s}} = \left[ {\matrix{ {{{\bf A}_{\bf s}}} \cr {{\bf B}_{\bf s}^{\rm \prime }} \cr } } \right].$$

The output network includes *m*_1_ directed edges among *m* gene and *n*_1_ directed edges from *n* NLTs to *m* genes.

Note that we have initially applied the GGM in our previous work to the whole matrix **M** directly (Wang et al., 2016). However, since the number of NLTs is much larger than that of genes, numerical results showed that the majority of selected edges connect NLTs, but few edges are selected to connect genes. This result is not appropriate because the regulations between genes should be the primary mechanisms of the network. Then we conducted another test, in which we did not consider the regulations between NLTs by changing matrix **C** into an identity matrix **I**_m_. Matrix **M** now is

(4) $${{\bf M}_1} = {\rm }\left[ {\matrix{ {{{\bf A}_{\bf s}}} & {\bf B} \cr {{\bf B}^{\prime }} & {{{\bf I}_{\bf m}}} \cr } } \right].$$

However, when we applied the GGM to **M**_1_ directly, the singular problem arose during the computation of IMLEA. To satisfy our intention and make the algorithm stable, we proposed EFSA which is executed in two steps. The first step selects regulations between genes and the second step finds regulations from NLTs to genes. The EFSA can be used to predict the gene-gene interactions and the effect from NLTs to genes based on the time-course experimental data.

### Bottom-up approach: mathematical model

For a regulatory network with *m* genes, the expression levels of the *i*-th gene at time *t* is denoted as *x*_*i*_(*t*). We used the following ordinary differential equation (ODE) model to describe the dynamics of the network (de Jong, 2002)

(5) $$\displaystyle{{d{\bf x}} \over {dt}} = F(t,{\bf x}),$$

where **x** = (*x*_1_,…,*x*_*m*_) is a vector representing the expression levels of *m* genes. A number of mathematical formalisms have been proposed to describe the dynamical interactions between different genes in the network, such as the models with linear functions (de Jong, 2002)

(6) $${F_i}(t,{\bf x}) = \sum\limits_{j = 1,j \ne i}^n {{a_{ij}}} {x_j} - {k_i}{x_i}$$

or the models with non-linear functions (Olariu & Peterson, 2018)

(7) $${F_i}(t,{\bf x}) = \displaystyle{{\sum\nolimits_{j = 1}^n {{a_{ij}}} {x_j}} \over {1 + \sum\nolimits_{j = 1}^n {{b_{ij}}} {x_j}}} - {k_i}{x_i}$$

The advantage of the model (Eq. 5) with the linear functions (Eq. 6) is that it has a much smaller number of unknown parameters than the non-linear functions (Eq. 7). However, the non-linear model is able to describe the non-linear dynamics more precisely. Therefore, we proposed a method that combines the feature of additive terms in the linear model and the advantages of non-linear model. We applied the second truncated Taylor series approach to approximate the non-linear function (Eq. 7). Here the Taylor series is a mathematical formula to approximate a function by using a polynomial function (Stewart, 2018). Thus, we proposed an ODE model (Eq. 5) with the following functions

(8) $${F_i}(t,{\bf x}) = \sum\limits_{j = 1,j \ne i}^m {{\rm \alpha _{ij}}} {x_j} + \sum\limits_{1 \le j \lt k \le n} {{\rm \beta _{ijk}}} {x_j}{x_k} - {k_i}{x_i}$$

where *k*_*i*_ is the degradation rate of *x*_*i*_. This proposed model (Eq. 5) with the non-linear function (Eq. 8) is based on the following assumptions:

1. The regulations from different genes to a particular gene are additive. Similarly, the regulations from non-linear terms (NLTs) to a particular gene are also additive.
2. The regulations from gene *j* to gene *i* is represented by α_*ij*_*x*_*j*_, where α_*ij*_ is the coefficient of regulation strength.
3. The regulation of NLT *x*_*j*_*x*_*k*_ to gene *i* is represented by β_*ijk*_*x*_*j*_*x*_*k*_, where β_*ijk*_ consists of the regulation strength and equilibrium constant *C*_*ij*_, as we discussed in the sub-section Top-down Approach.
4. The auto-regulation is not considered, namely α_*ii*_ = 0, to avoid confusion between auto-regulation term α_*ii*_*x*_*i*_ and degradation term *k*_*i*_*x*_*i*_. Note that the issue of auto-regulation may be addressed using a model with non-linear function (Eq. 7). In addition, we just consider the effect of NLTs *x*_*j*_*x*_*k*_ for *j* ≠ *k* since the expression levels of *x*_*j*_ may be highly correlated to that of $x_j^2$. Therefore, we assume that β_*ijj*_ = 0.
5. If the value of α_*ij*_ is positive (negative or zero), it means that gene *x*_*j*_ activates (represses or has no regulation to) the expression of gene *x*_*i*_. Similar assumption is applied to the value of β_*ijk*_.

We emphasize that the proposed method in this work is substantially different from our previous work (Wang et al., 2016). The first difference is that the proposed non-linear model (Eq. 8) is different from the non-linear model in Wang et al. (2016). This new model not only can study the regulations from genes to genes, as we considered in our previously proposed model (Wang et al., 2016), but also can investigate the effects of heterodimers and/or synergistic effect in genetic regulation. This new model also leads to the second difference compared with our previous top-down approach, namely the proposed Extended Forward Search Algorithm (EFSA) not only includes the probabilistic graphical model in our previous work (Wang et al., 2016) but also can predict the possible regulations from NLTs to genes. In addition, in this work, we will infer a medium-sized network first by using EFSA and then reduce the network size by removing regulations from the network in the Results section, rather than inferring a core network first and then adding regulations to the core network in our previous approach (Wang et al., 2016).

### Parameter inference

When considering the full connected graph among *m* genes and *n* non-linear terms (NLTs), we have an ordinary differential equation (ODE) system with *m* differential equations. The total number of all unknown coefficients is *m*(*m* + *n*). After applying the Extended Forward Search Algorithm (EFSA), we have an inferred regulatory network which contains only *m*_1_ edges among genes and *n*_1_ edges from NLTs to genes. Thus, the numbers of coefficients α_*ij*_ and β_*ijk*_ are reduced from *m*(*m −* 1) to *m*_1_ and from *mn* to *n*_1_, respectively. It is easier to estimate the parameters for the inferred network than for the fully connected network.

In this work, we used a MATLAB toolbox of Genetic Algorithm to estimate the parameters in the proposed mathematical model (Chipperfield, Fleming & Fonseca, 1994). The algorithm begins by generating a population of initial parameter values, for example, 100 values. Each initial value is called an individual and the whole population is called one generation. Then it calculates the fitness value for each individual of current generation. Based on the fitness values, the algorithm next creates new values for each individual and thus forms a population of the next generation. This process is repeated until a pre-defined number of generations have been calculated. In this work, we used the following functions, namely function *crtbp* to generate initially binary populations, function *reins* to effect fitness-based reinsertion, function *select* to give a convenient interface to the selection routines, function *recombine* to conduct crossover operators, and function *mut* to conduct binary and integer mutations. The detailed information of these functions and their alternatives can be found in the relevant reference (Chipperfield, Fleming & Fonseca, 1994).

To ensure the accuracy of estimates, we set the number of generations as 1,000 and the number of individuals for each generation as 300. For the parameter vector (α_*ij*_, β_*ijk*_, *k*_*i*_), we used the uniform distribution over the interval (*W*_min_, *W*_max_) to generate the initial estimates. Here *W*_min_ and *W*_max_ are the minimal value and maximal value, respectively, for choosing the samples of the parameters. The values of *W*_min_ and *W*_max_ are adjusted by computation. For example, if the majority of estimated parameters all are close to *W*_min_, then we will further decrease the value of *W*_min_. However, if the majority of estimated values are well above *W*_min_, then we need to increase the value of *W*_min_ accordingly. The similar consideration is applied to *W*_max_. In this study, for the erythroid lineage pathway, numerical results suggest that the values of *W*_min_ and *W*_max_ for (α_*ij*_, β_*ijk*_, *k*_*i*_) are ( −3, −3, 0) and (3, 3, 1), respectively. In addition, for the neutrophil lineage pathway, numerical results suggest that the values of *W*_min_ and *W*_max_ for (α_*ij*_, β_*ijk*_, *k*_*i*_) are (−2.5, −2.5, 0) and (2.5, 2.5, 1), respectively. We run the algorithm using an initial random number to generate an initial set of model parameters, which leads to a set of estimated parameters. For each model, we used 200 different initial random numbers, which lead to 200 different sets of estimated model parameters. Denote *x*_*i*_(*t*_*j*_) and x*_i_(*t*_*j*_) as the observation data and numerical simulations at time point *t*_*j*_ for *j* = {1,2,…,*M*}, respectively. The simulation error is calculated by

(9) $$E = \sqrt {\sum\limits_{i = 1}^m {\sum\limits_{j = 1}^M {({x_i}(} } {t_j}) - x_i^*({t_j}){)^2}}.$$

We selected the top ten sets with the minimal estimated errors out of 200 estimates for further analysis and comparison.

#### Robustness analysis

We noted that, if a model with the estimated parameters is not robust, a perturbation to the parameters might lead to substantial variations of the model output. Thus, we next used the robustness property of the model to select the inferred model parameter sets from the Genetic Algorithm. This property was designed to examine the robustness of the inferred model to the perturbations of model parameters (Kitano, 2004). Robustness property is also an important method for understanding the variations in genetic regulatory networks mathematically (Masel & Siegal, 2009). Note that our perturbation test is a mathematical technique. It is different from the perturbation of biological experiments, which may be conducted by the over-expression/knock-down tests. Although we will conduct removal tests by removing edges from the developed model, these tests are designed to remove the unnecessary (or unimportant) regulations in the network.

In this perturbation test, based on the inferred parameter *k*_*i*_ that is assumed to be the unperturbed one, the perturbed parameter is generated by

(10) $$\overline {{k_i}} = {k_i} \times (1 + {\rm \mu} \times {\rm \varepsilon})$$

where ε is a sample generated from either the normal distribution or the uniform distribution. In this work, we used the standard Gaussian random variable **N**(0,1) to generate samples. In addition, μ is a parameter to determine the values of perturbation (Wang et al., 2016). The value of parameter μ determines the variations of simulations. Numerical results suggest that when the value of μ is small, perturbation has small effect on the system dynamics, and it is difficult to distinguish the robustness properties of the model with different parameter sets. However, if the value of μ is large, perturbation will make the model output substantially different, and it will be difficult to measure the robustness property. To make the variations of simulations appropriately for robustness analysis, μ = 0.4 was employed in this study.

For each of the top ten sets of parameters determined in the previous sub-section, we firstly obtained *N* ( = 5,000) sets of perturbed model parameters by using (Eq. 10) and then used these parameter sets to obtain *N* corresponding simulations. We used $x_{ij}^{(k)}(p)$ and $x_{ij}^{(k)}$ to denote the simulation of variable *x*_*i*_ at time point *t*_*j*_ obtained by the *k*-th perturbed and unperturbed model parameters, respectively. Then, we defined

(11) $${E^{(k)}} = \sqrt {\sum\limits_{i = 1}^m {\sum\limits_{j = 1}^M {(x_{ij}^{(k)}(} } p) - x_{ij}^{(k)}{)^2}}$$

as the measure for the robustness property of the model with the *k*-th perturbed parameter set. Afterwards, we defined the robust average for the given parameter set as

(12) $$RA = \displaystyle{1 \over N}\sum\limits_{k = 1}^N {{E^{(k)}}},$$

and robust standard deviation as

(13) $${RSTD} = \sqrt {\displaystyle{1 \over {N - 1}}\sum\limits_{k = 1}^N {{{({E^{(k)}} - {RA})}^2}}}$$

over *N* perturbation tests. Smaller values of RA and RSTD mean that the model with the given parameter set is more robust.

## Results

### Inference of regulatory network

To reduce the complexity of regulatory networks, we first used the Extended Forward Search Algorithm (EFSA) to predict the topological structure of genetic networks. The algorithm controls the number of edges by adjusting a pre-defined cut-off value. This value is equivalent to the significant value in statistics. If the threshold is too low, we may miss some significant regulations. However, if the threshold is relatively high, it is quite possible to select insignificant regulations. This work considers the networks including 11 genes and 55 non-linear terms (NLTs). For the sub-network of 11 genes only (i.e. matrix **A**_**s**_ in Eq. 3), to ensure the statistically significant, we set a specific threshold as 0.1 for both the erythroid regulatory network and neutrophil regulatory network. The selection of this threshold value (i.e. 0.1) is based on the balance between neither selecting much insignificant regulations nor choosing a small number of candidate regulations. Then we had 46 and 40 directed edges for the erythroid regulatory network and neutrophil network, respectively.

For the regulations from NLTs to genes (i.e. matrix **B**′_**s**_ in Eq. 3), the size of matrix **B**′_**s**_ is much larger than that of **A**_**s**_. To avoid the dominance of the regulations from NLTs to genes, we also set the cut-off value as 0.1 for the two networks, or take the first 46 and 40 directed edges from NLTs to genes for the erythrocyte and neutrophil differentiation, respectively, if more edges are selected when using the cut-off value 0.1. The reason we still applied threshold 0.1 here is that the number of selected edges that satisfy this value is much larger than the required number (i.e. 46 for the erythroid regulatory network and 40 for the neutrophil regulatory network). Since the edges are selected and ranked by their significance, we can simply select the top 46 edges and 40 edges for the erythroid and neutrophil pathway, respectively, without conducting any further numerical tests.

Figures S1 and S2 in Supplemental Information present the inferred regulatory networks for the erythroid and neutrophil networks, respectively. Note that there are 11 and 17 isolated NLTs in the erythroid and neutrophil networks, respectively, since no significant edges have been selected from these NLTs by our algorithm. All these isolated NLTs are listed in the “Isolated NLTs Table”. Moreover, all arrows in these figures only represent the direction of regulations, rather than the types of regulations (i.e. positive or negative regulation). We will study the detailed regulatory mechanisms in the next subsection. We found that the targeted gene of the protein heterodimer is a component of that heterodimer in all situations. The possible explanation of this observation is that the expression levels of a heterodimer are the product of the expression levels of the two corresponding genes (namely *x*_*i*_*x*_*j*_ for genes *i* and *j* with expression levels *x*_*i*_ and *x*_*j*_, respectively). Thus, the expression data of the NLTs {*x*_*i*_*x*_*j*_} may be highly correlated to those of the component genes, namely {*x*_*i*_} or {*x*_*j*_}.

### Inference of mathematical model

After the success of constructing regulatory networks in the previous sub-section, we next study the detailed dynamics of genetic networks in fate determination of hematopoietic stem cells (HSCs) by using our proposed mathematical model. The major step is to infer the values of unknown parameters in the model (Eq. 8). If we consider the fully connected model, there should be 11 × (11 + 55) = 726 parameters. However, after the application of EFSA, the number of unknown parameters is reduced to 103 (including 46 directed edges between genes, 46 directed edges from non-linear terms (NLTs) to genes and 11 self-degradation rate constants) for the differentiation of erythrocytes and 91 (including 40 directed edges between genes, 40 directed edges from NLTs to genes and 11 self-degradation rate constants) for the differentiation of neutrophils. We next applied the Genetic-Algorithm to estimate these unknown parameters for two networks. We used 200 different random numbers to obtain different initial values of rate constants (α_*ij*_, β_*ijk*_, *k*_*i*_) over the defined range (*W*_min_, *W*_max_), which was discussed in the Methods section. This leads to 200 different sets of estimated parameters. Then, we chose the top ten sets of estimated results for each differentiated lineage with the smallest estimation errors for further robustness analysis. According to the definition of estimation error (Eq. 9), the optimal inferred network for the erythrocyte differentiation in our tests has estimation error 0.9902. In addition, the robust average (Eq. 12) and robust standard deviation (Eq. 13) are 0.3977 and 0.1066, respectively. For the neutrophil differentiation, the optimal inferred network has estimation error 0.8726, robust average 0.3983 and robust standard deviation 0.1275.

Figures 1 and 2 present the simulation results based on the optimal estimated parameters for the expression levels of four genes, namely genes *Gata1, PU.1, Ets1* and *Tal1*, for the differentiation of erythrocyte and neutrophil, respectively. The expression levels of *Gata1* increase continuously in both simulated and experimental data during the erythrocyte differentiation. However, during the neutrophil differentiation, experimental data of *Gata1* keep fluctuations and then turn to slightly decreasing at the end of differentiation, which is matched by our simulation. For gene *PU. 1*, both microarray and simulated data decline in the differentiation of erythrocyte but climb during the differentiation of neutrophil. Similarly, the expression levels of *Ets1* in microarray data increase during erythrocyte differentiation but decrease during neutrophil differentiation. Simulation results also fit the trends for both differentiation pathways. The experimental data of *Tal1* increase with fluctuations during the first 60 h of erythrocyte differentiation, but then rises rapidly after the first 60 h. Our simulated results are consistent with the expression levels of *Tal1* with the same trend in expression levels. Thus, our simulation results fit the trend of expression levels of these genes very well during two developmental processes. Figures S3 and S4 in Supplemental Information give the simulation results of the other six genes for the differentiation of erythrocyte and neutrophil, respectively.

### Reduction of network model—edge deletion

We have obtained two regulatory networks with 92 directed edges and 80 directed edges for erythroid and neutrophil differentiation, respectively. Next we tested the possibility to delete the potential insignificant edges from our predicted regulatory networks. In the first step, we tested the deletion of regulations from non-linear terms (NLTs) to genes. We removed one edge in each test to form a temporary system model, and then examined the simulation error and robustness property of the new model. Afterwards, we removed one specific edge permanently if the corresponding new system has the minimal change in simulation error and robustness property, and then formed an updated model. This test is repeated until both the simulation error and robustness property of the updated model are much worse than the original network without any removal. In the second step, we evaluated the regulatory interactions between 11 genes using the same method in the first step.

For the erythrocyte differentiation, Table 1 suggests that after removing 3 regulations from NLTs to genes, the estimation error (Eq. 9) is improved (shown in DEL1). Then, we tested the regulation reduction from gene to gene. The final result suggests that, after we deleted (*Ldb1* → *Lmo2*), (*Notch1* → *Lmo2*), (*Cbfa2t3* → *Lmo2*) and (*Runx1* → *Lmo2*) edges, the estimation error (Eq. 9) is slightly increased. However, the robustness property is better than that of the DEL1 model since the robust average (Eq. 12) is decreased. Thus, for the erythroid differentiation, numerical tests recommended to remove total seven edges from our predicted regulatory network. We stopped the deletion test after obtaining the DEL5 model. If we proceed further deletion, both simulation error and robustness property of the temporary network are much worse than the original network without removal.

**Table 1:** Edge deletion test for erythrocyte differentiation. RR, Removed regulation; SE, Simulation error, defined by Eq. (9); RA, Robust average, defined by Eq. (12); RSTD, Robust standard deviation, defined by Eq. (13).

Model	RR	SE	RA	RSTD
OES	N/A	0.9902	0.3977	0.1066
DEL1	Gata2-Notch1 *→* Notch1 Tal1-Gfi1 *→* Gfi1 Cbfa2t3-Gfi1 *→* Gfi1	0.9826	0.4594	0.1259
DEL2	Ldb1 *→* Lmo2	0.9955	0.3938	0.1124
DEL3	Notch1 *→* Lmo2	0.9861	0.4506	0.1263
DEL4	Cbfa2t3 *→* Lmo2	1.0451	0.3820	0.0962
DEL5	Runx1 *→* Lmo2	1.0298	0.3471	0.0904

**Note:**

Description of different models: OES, The original model without any deletion; DEL1, Model based on OES by removing regulations from NLTs to genes; DEL2, Model based on DEL1 by removing a regulation among genes; DEL3, Model based on DEL2 by removing a regulation among genes; DEL4, Model based on DEL3 by removing a regulation among genes; DEL5, Model based on DEL4 by removing a regulation among genes.

Table 2 shows that, for the neutrophil differentiation, there are no insignificant regulations from NLTs to genes, because the removal of any edge from NLTs to genes will increase the simulation error (Eq. 9) substantially and/or decrease the robustness property by increasing the values of robust average (Eq. 12) and robust standard deviation (Eq. 13). For the regulations between genes, we have removed the following four regulations, namely (*Gata2* → *Ldb1*), (*Runx1* → *Cbfa2t3*), (*Ldb1* → *Lmo2*) and (*Tal1* → *Lmo2*), and formed an updated system. Table 2 shows that the simulation error and robustness property of the updated system are close to those of the original system without any removal of edges. Thus, for the neutrophil differentiation, numerical tests recommended to remove only four edges from our predicted regulatory network. Coincidentally, we stopped the deletion test after obtaining the DEL5 model because of the same reason for the erythrocyte differentiation.

**Table 2:** Edge deletion test for neutrophil differentiation. RR, Removed regulation; SE, Simulation error, defined by Eq. (9); RA, Robust average, defined by Eq. (12); RSTD, Robust standard deviation, defined by Eq. (13).

Model	RR	SE	RA	RSTD
OES	N/A	0.8726	0.3983	0.1275
DEL1	No Suggestion	N/A	N/A	N/A
DEL2	Gata2 *→* Ldb1	0.8726	0.3943	0.1273
DEL3	Runx1 *→* Cbfa2t3	0.8726	0.3928	0.1265
DEL4	Ldb1 *→* Lmo2	0.8748	0.4183	0.1333
DEL5	Tal1 *→* Lmo2	0.8809	0.3925	0.1237

**Note:**

Description of different models: OES, The original model without any deletion; DEL1, Model based on OES by removing regulations from NLTs to genes; DEL2, Model based on DEL1 by removing a regulation among genes; DEL3, Model based on DEL2 by removing a regulation among genes; DEL4, Model based on DEL3 by removing a regulation among genes; DEL5, Model based on DEL4 by removing a regulation among genes.

**Figure 3:**
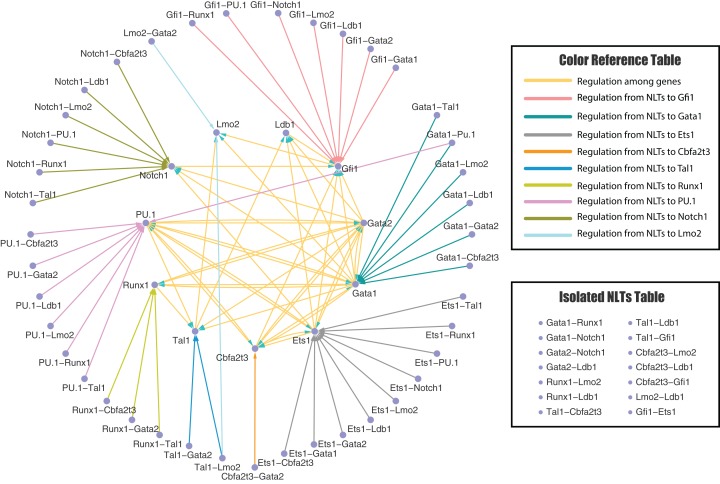
Predicted genetic regulatory network of erythrocyte pathway. The genetic regulatory network predicted by the Extended Forward Search Algorithm with 11 genes and 41 non-linear terms (NLTs) (14 isolated NLTs excluded) after edges deletion test, which is related to the fate determination of erythrocyte pathway: regulatory network for hematopoietic stem cells differentiate to megakaryocyte-erythroid progenitors. The network is visualized by Cytoscape software.

**Figure 4:**
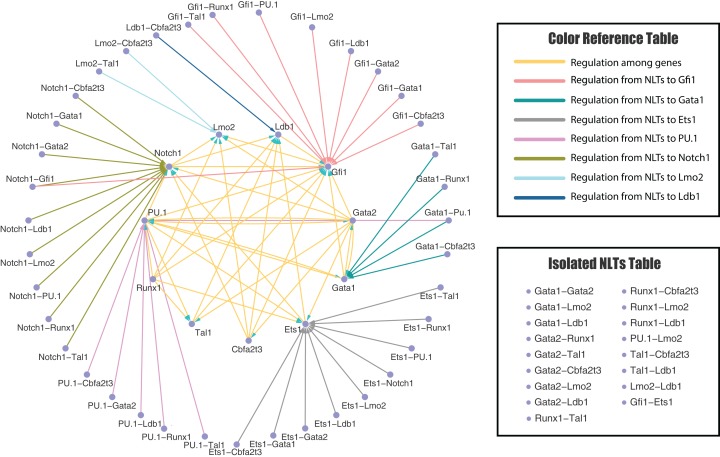
Predicted genetic regulatory network of neutrophil pathway. The genetic regulatory networks predicted by the Extended Forward Search Algorithm with 11 genes and 38 non-linear terms (NLTs) (17 isolated NLTs excluded) after edges deletion test, which is related to the fate determination of neutrophil pathway: regulatory network for hematopoietic stem cells differentiate to granulocyte-macrophage progenitors. The network is visualized by Cytoscape software.

Figures 3 and 4 present the inferred regulatory networks after edge deletion test for erythroid and neutrophil differentiation, respectively. Initially, we have 92 directed edges for the erythrocyte pathway and 80 directed edges for the neutrophil pathway. After the edges deletion, seven and four directed edges have been taken away from the erythrocyte network and neutrophil network, respectively, since the removal of these edges has not much negative influence on simulation error (Eq. 9), robust average (Eq. 12) and robust standard deviation (Eq. 13). Thus, there are 85 and 76 directed edges left for the erythrocyte and neutrophil pathways, respectively.

## Discussion

This work was designed to develop a mathematical framework that was able to realize nonlinear gene expression dynamics accurately. In particular, we intended to investigate the effect of possible protein heterodimers and/or synergistic effect in genetic regulation. In this study, we designed the Extended Forward Search Algorithm (EFSA) to predict the topology of regulatory networks connecting genes and heterodimers. We also proposed a new mathematical model for inferring dynamic mechanisms of regulatory networks. Using the EFSA, we derived two regulatory networks of 11 genes for erythrocyte and neutrophil differentiation pathways. According to the predicted networks and experimental data, we estimated parameters in our proposed mathematical model based on the criteria of simulation error and robustness property. By removing regulations with less importance based on simulation error and robustness property, we developed two gene networks that regulate erythrocyte and neutrophil differentiation pathways. Numerical results suggested that our proposed method is capable of reconstructing genetic regulatory networks effectively and accurately.

To infer the regulatory mechanisms of heterodimers, we combined both the top-down approach (i.e. probabilistic graphical model) and the bottom-up approach (i.e. mathematical model). We used the top-down approach first to simplify the network topology and reduced the number of unknown parameters in the mathematical model. Then the Genetic-Algorithm was used to estimate the unknown parameters. The combination of these two approaches reduced the errors in simulation and also improved the robustness property of the mathematical model. In this work, we considered the network with medium-sized complexity initially. We then reduced the network complexity by removing edges from the network, rather than studying the core network and then adding the edges to the network in our previous study (Wang et al., 2016). The reason for changing the method from “adding edge” to “removing edge” in this work is mainly due to the high computational cost in the “adding edge” tests since the number of candidate edges in the “removing edge test” is much smaller than that in the “adding edge test”. Thus, in this work, we used the EFSA to obtain more candidate edges and then used the dynamic model to remove unimportant edges. If the number of potential regulations derived from the probabilistic graphical model is relatively large, the removal of one single regulation from the potential network may not have any changes in simulation error. Numerical results suggested that a couple of regulations should be removed simultaneously in order to achieve changes in simulation error.

The inferred regulatory networks from our proposed models are partially supported by experimental observations. For example, the regulation of *Gata1-Gata2-PU.1* complex in our inferred networks agrees with the experimental results (May et al., 2013). The *Gata1-PU.1* heterodimer plays an important role in regulating the hematopoiesis (Zhang et al., 2000), which is also included in our inferred model. In addition, the *Ldb1-Lmo2* dimer is activated with significantly expression profiles during the erythroid differentiation process (Xu et al., 2003), which is consistent with our prediction. Moreover, there are evidences to show the existence of synergistic effect of *Tal1, Lmo2* and *Gata1* (Mead et al., 2001), which has been inferred in our regulatory networks as well. However, not all of the predictions can be confirmed by the existing experimental observations. The first explanation is that the non-linear terms in our mathematical model are introduced by mathematical operation (i.e. the Taylor series). Some of these non-linear terms may be needed for realizing the nonlinear dynamics accurately, but not supported by biological mechanisms. Note that another inference method, called semi-supervised method, can include the validated regulations first and then infer the invalidated regulations (Maetschke et al., 2013). Secondly, our inferred regulatory network may predict some potential possible regulations between genes and from non-linear terms to genes, which may be confirmed by future experimental studies. Thus, the inferred regulations in this work may provide testable prediction for further experimental studies to explore the detailed mechanism of hematopoiesis.

This work also raised a number of important issues in the study of genetic regulations. One question is that our non-linear model still cannot fit all the expression data very well due to noise in the data. Figures 1 and 2 show that the noise in expression data may increase the simulation error of our proposed model. If the noise ratio in expression data is large, it is a challenging issue in mathematical modeling. Large variations in the data may lead to incorrect inference results. In that case, stochastic modeling may be a more appropriate approach to describe the noise in gene expression data (Samad et al., 2005; Tian, 2010; Chowdhury, Chetty & Evans, 2015). In addition, the Gaussian graphical model is based on the covariance matrix. However, the correlation coefficient is suitable to measure the linear correlation relationship. Currently, other approaches, such as mutual information and conditional mutual information, have been used to measures both linear and non-linear correlation relationships between the gene expression data (Zhang et al., 2012, 2015; Zhao et al., 2016). Finally, this research determines the regulatory mechanisms based on numerical simulation and robustness property. More information from experimental studies will be important to improve the accuracy of the model and make more reasonable predictions. In addition, we may use other key criteria to select mathematical models, such as Akaike’s Information Criterion (AIC), Bayesian Information Criterion (BIC), and Bayesian factor (Kadane & Lazar, 2004). All these issues will be the interesting topics of our future research.

## Conclusion

In conclusion, this study proposes a new method to construct the network topology from genes and heterodimers by a new top-down approach and then develops a non-linear ordinary differential equation model to infer the dynamic mechanisms of regulatory networks. The derived two networks may provide insights regarding the genetic regulations in the cell fate determination of hematopoietic stem cells. The proposed method can also be applied to model other regulatory pathways and biological systems.

## Supplemental Information

10.7717/peerj.9065/supp-1 Supplemental Information 1 Name list of selected 30 genes.

## Additional Information and Declarations

### Data Availability

The following information was supplied regarding data availability: The data is available at NCBI GEO: GSE49991. The code is available at GitHub: https://github.com/ThaddeusWu/PeerJ_Submission.
