# Supplementary material for: A non-linear reverse-engineering method for inferring genetic regulatory networks
Source: PeerJ. 2020 Apr 29;8:e9065. doi: 10.7717/peerj.9065 (PMC7195839; doi:10.7717/peerj.9065)
Supplement: Supplemental Information 1 [file peerj-08-9065-s001.pdf]

Supplementary Information:

A Non-linear Reverse-engineering Method for Inferring Genetic Regulatory Networks

Siyuan Wu, Tiangang Cui, Xinan Zhang and Tianhai Tian

1. Inferred regulatory network for the differentiation of erythrocyte by EFSA

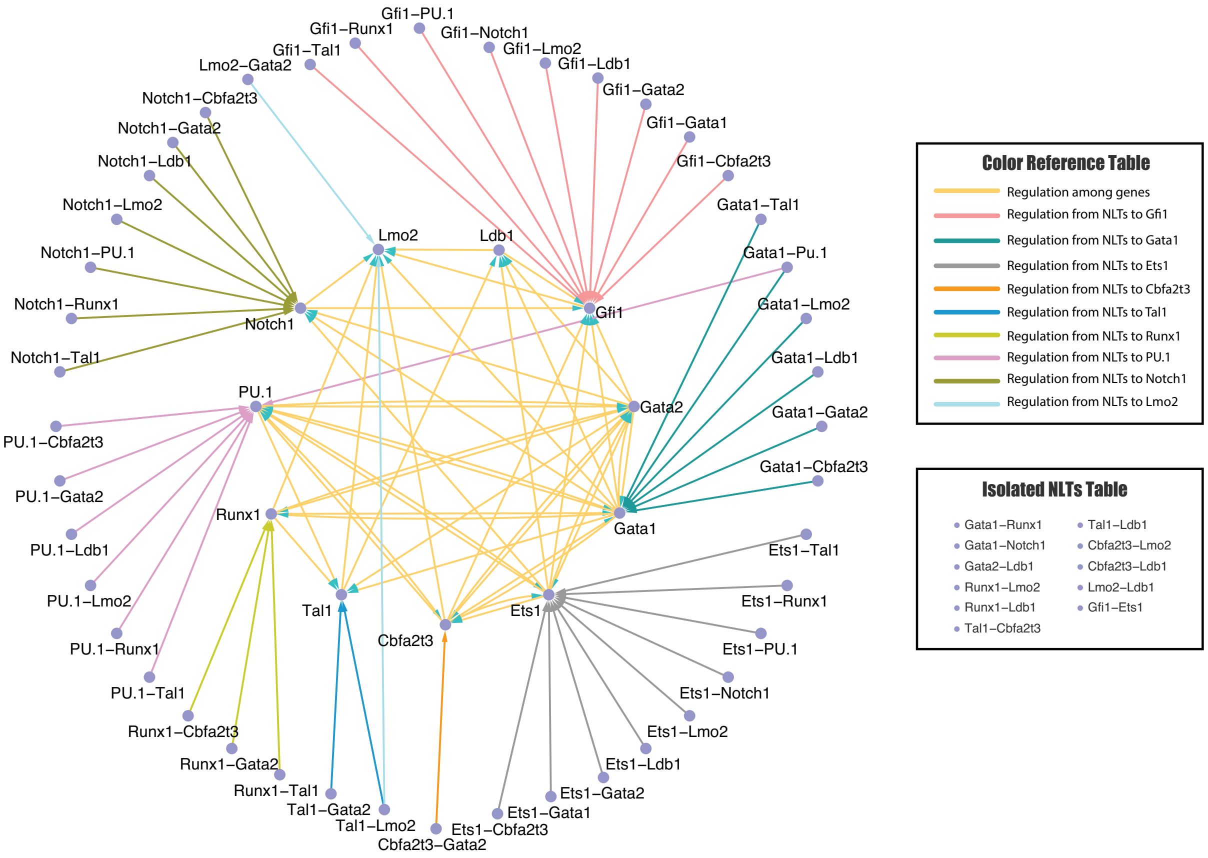

SFigure 1. Inferred network structure before edges deletion. The genetic regulatory network predicted by EFSA with 11 genes and 44 NLTs (11 isolated terms excluded), which is related to the fate determination of erythrocyte pathway: Regulatory network for hematopoietic stem cells differentiate to megakaryocyte-erythroid progenitors. The network is visualized by Cytoscape software.

2. Inferred regulatory network for the differentiation of neutrophil by EFSA

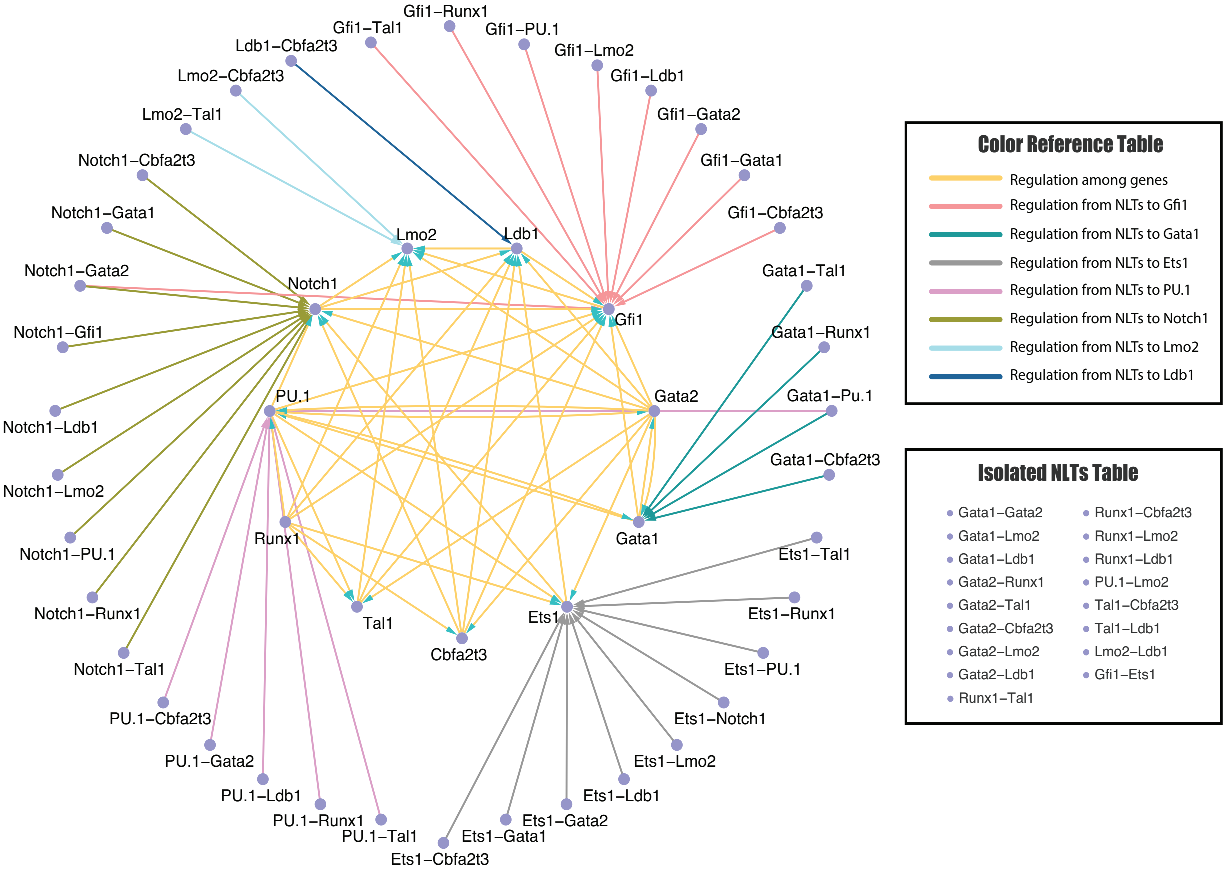

SFigure 2. Inferred network structure before edges deletion. The genetic regulatory networks predicted by EFSA with 11 genes and 38 NLTs (17 isolated terms excluded), which is related to the fate determination of neutrophil pathway: Regulatory network for hematopoietic stem cells differentiate to granulocyte-macrophage progenitors. The network is visualized by Cytoscape software.

3. Simulation results and experimental data of the regulatory network for the erythrocyte differentiation

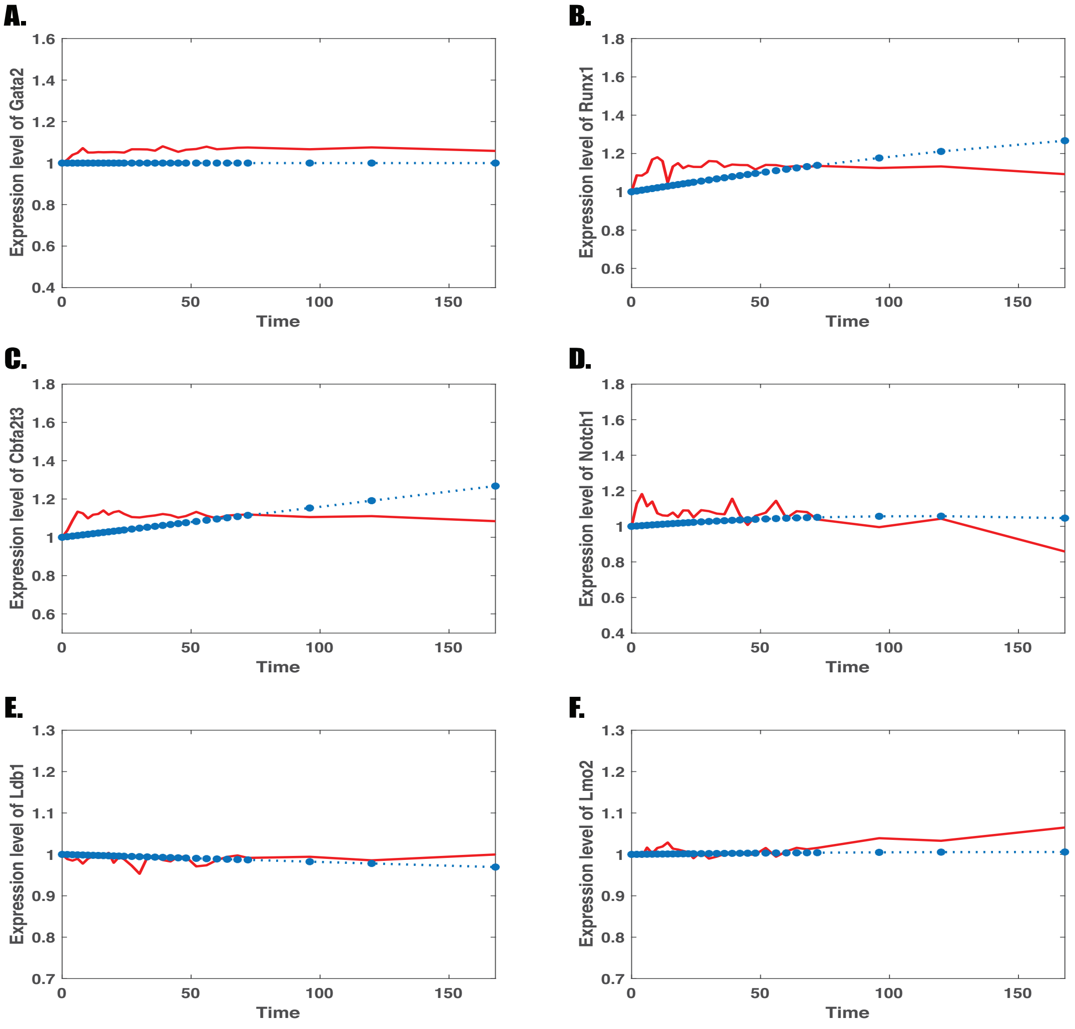

SFigure 3. Simulation results and experimental data of the regulatory network for the erythrocyte differentiation. (Red solid line: experimental microarray data; Blue star dash line: simulation of the regulatory network).

4. Simulation results and experimental data of the regulatory network for the neutrophil differentiation

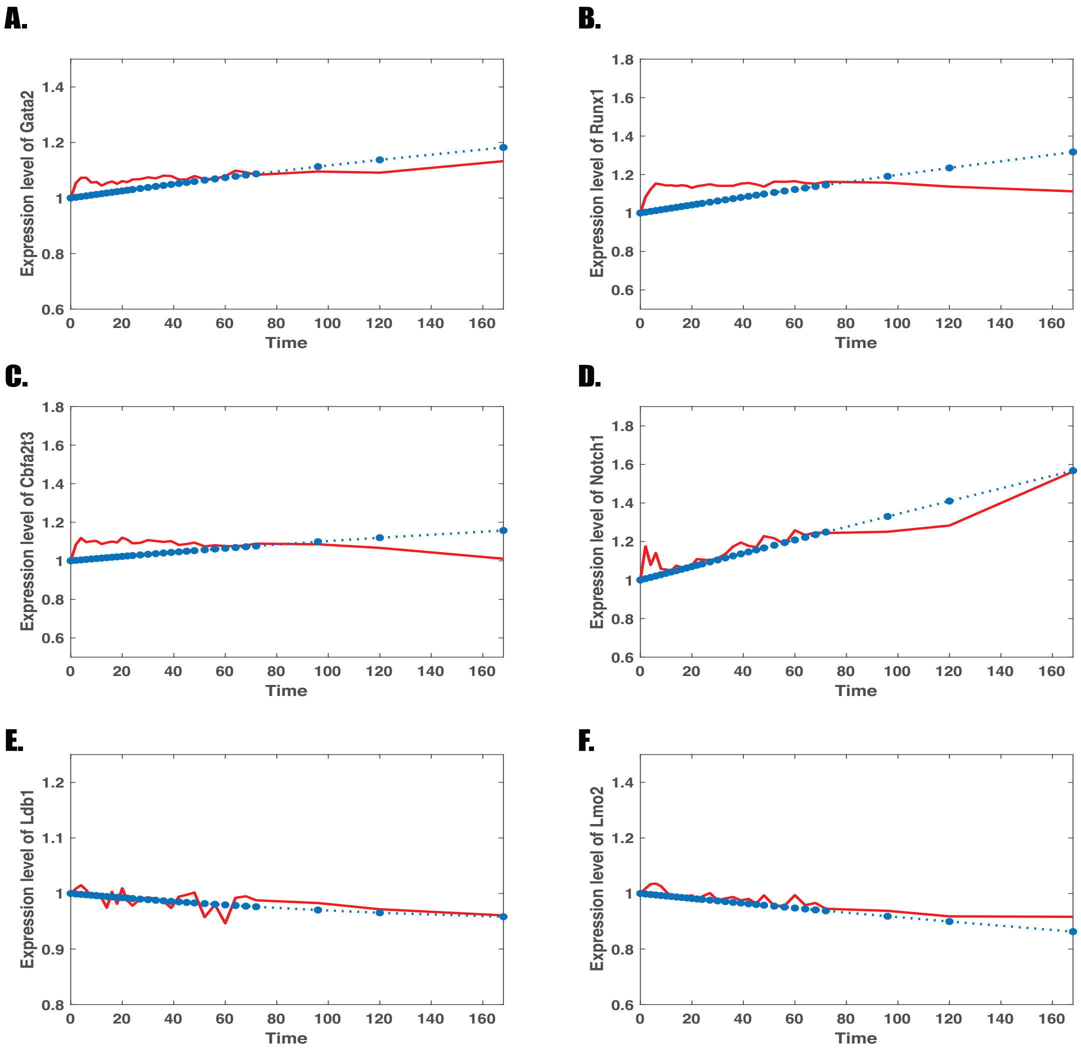

SFigure 4. Simulation results and experimental data of the regulatory network for the neutrophil differentiation. (Red solid line: experimental microarray data; Blue star dash line: simulation of the regulatory network).

5. Selection of 30 candidate genes for differentiation of hematopoietic stem cells

STable 1: Information of the 30 candidate genes for differentiation of hematopoietic stem cells. The 30 genes in "This study" are the combination of the genes in two published studies.

Reference Paper	Gene number	Gene name
**Moignard et al., 2013, Figure 1**	18	Gata1, Gata2, PU.1, Gfi1, Gfi1b, Hhex, Ldb1 Lmo2, Lyl1, Meis1, Mitf, Nfe2, Runx1, Tal1, Etv6, Erg, Cbfa2t3
**Moignard et al., 2015, Figure 3**	26	Mesi1, Mitf, Etv2, Fli1, Tal1, Gata1, Hoxb4, Lyl1, Notch1, Sox7, PU.1, Ets1, Erg, Nfe2, Cbfa2t3, Lmo2, Myb, Hoxb2, Sox17, Gfi1, Gfi1b, Hhex, Tbx3, Tbx20, FoxH1, Ikaros
**This study**	30	Gata1, Gata2, PU.1, Gfi1, Gfi1b, Hhex, Ldb1 Lmo2, Lyl1, Meis1, Mitf, Nfe2, Runx1,Tal1, Etv6, Erg, Cbfa2t3, Etv2, Fli1, Hoxb4, Notch1, Sox7, Ets1, Hoxb2, Sox17, Tbx3, Tbx20, FoxH1, Ikaros, Myb

References

1. Moignard, V., Macaulay, I. C., Swiers, G., Buettner, F., Sch¨utte, J., Calero-Nieto, F. J., Kinston, S., Joshi, A., Hannah, R., Theis, F. J., Jacobsen, S. E., de Bruijn, M., and G¨ottgens, B. (2013). Characterization of transcriptional networks in blood stem and progenitor cells using high-throughput single-cell gene expression analysis. Nat Cell Biol, 15(4):363–372.
2. Moignard, V., Woodhouse, S., Haghverdi, L., Lilly, A. J., Tanaka, Y., Wilkinson, A. C., Buettner, F., Macaulay, I. C., Jawaid, W., Diamanti, E., Nishikawa, S.-I., Piterman, N., Kouskoff, V., Theis, F. J., Fisher, J., and G¨ottgens, B. (2015). Decoding the regulatory network of early blood development from single-cell gene expression measurements. Nat Biotechnol, 33(3):269–279

6. Literature review for the selection of 11 important genes

Stable 2. Literature information for the selected 11 genes in this study.
These genes are selected from STable 1 based on their relationship with the three genes Gata1, Gata2 and PU.1. The details of information is also described in the Methods section of the paper. Here is the the summary table.

Gene Name	Reference
**Gata1**	Friedman, 2007; Liew et al., 2006; Ling et al., 2004
**Gata2**	Friedman, 2007; Liew et al., 2006; Ling et al., 2004
**PU.1**	Friedman, 2007; Liew et al., 2006; Ling et al., 2004
**Runx1**	North et al., 2004
**Cbfa2t3**	Goardon et al., 2006
**Ets1**	Lulli et al., 2006
**Notch1**	Kumano et al., 2001; Stier et al., 2002
**Tal1**	Goardon et al., 2006; Shivdasani et al., 1995; Zhang et al., 2005; Porcher et al., 1996; Real et al., 2012
**Ldb1**	Soler et al., 2010; Li et al., 2011
**Gfi1**	North et al., 2004; van der Meer et al., 2010; Lancrin et al., 2012
**Lmo2**	Inouea et al., 2013; Visvader et al., 1997

References

1. Friedman, A. D. (2007). Transcriptional control of granulocyte and monocyte development. Oncogene, 26(47):6816–6828.
2. Goardon, N., Lambert, J. A., Rodriguez, P., Nissaire, P., Herblot, S., Thibault, P., Dumenil, D., Strouboulis, Romeo, P.-H., and Hoang, T. (2006). Eto2 coordinates cellular proliferation and differentiation during erythropoiesis. EMBO J, 25(2):357–366.
3. Inouea, A., Fujiwaraa, T., Okitsua, Y., Katsuokaa, Y., Fukuharaa, N., Onishia, Y., Ishizawaa, K., and Harigaea, H. (2013). Elucidation of the role of Lmo2 in human erythroid cells. Exp. Hematol, 41(12):1062–1076.
4. Kumano, K., Chiba, S., Shimizu, K., Yamagata, T., Hosoya, N., Saito, T., Takahashi, T., Hamada, Y., and Hirai, H. (2001). Notch1 inhibits differentiation of hematopoietic cells by sustaining gata-2 expression. Blood, 98(12):3283–3289.
5. Lancrin, C., Mazan, M., Stefanska, M., Patel, R., Lichtinger, M., Costa, G., Vargel, O¨ ., Wilson, N. K.,
6. Mo¨ro¨y, T., Bonifer, C., Go¨ttgens, B., Kouskoff, V., and Lacaud, G. (2012). Gfi1 and Gfi1b control the loss of endothelial identity of hemogenic endothelium during hematopoietic commitment. Blood, 120(2):314–322.
7. Li, L., Jothi, R., Cui, K., Lee, J. Y., Cohen, T., Gorivodsky, M., Tzchori, I., Zhao, Y., Hayes, S. M., Bresnick, E. H., Zhao, K., Westphal, H., and Love, P. E. (2011). Nuclear adaptor ldb1 regulates a transcriptional program essential for the maintenance of hematopoietic stem cells. Nat Immunol, 12(2):129–136.
8. Liew, C. W., Rand, K. D., Simpson, R. J. Y., Yung, W. W., Mansfield, R. E., Crossley, M., Proetorius-Ibba, M., Nerlov, C., Poulsen, F. M., and Mackay, J. P. (2006). Molecular analysis of the interaction between the hematopoietic master transcription factors gata-1 and pu.1. J Biol Chem, 281(38):28296–28306.

9. Ling, K. W., Ottersbach, K., van Hamburg, J. P., Oziemlak, A., Tsai, F. Y., Orkin, S. H., Ploemacher, R., Hendriks, R. W., and Dzierzak, E. (2004). Gata-2 plays two functionally distinct roles during the ontogeny of hematopoietic stem cells. J Exp Med, 200(7):871–872.
10. Lulli, V., Romania, P., Morsilli, O., Gabbianelli, M., Pagliuca, A., Mazzeo, S., Testa, U., Peschle, C., and Marziali, G. (2006). Overexpression of ets-1 in human hematopoietic progenitor cells blocks erythroid and promotes megakaryocytic differentiation. Cell Death Differ, 13(7):1064–1074.
11. North, T. E., Stacy, T., Matheny, C. J., Speck, N. A., and de Bruijn, M. F. (2004). Runx1 is expressed in adult mouse hematopoietic stem cells and differentiating myeloid and lymphoid cells, but not in maturing erythroid cells. Stem Cells, 22(2):158–168.
12. Porcher, C., Swat, W., Rockwell, K., Fujiwara, Y., Alt, F., and Orkin, S. H. (1996). The T cell leukemia oncoprotein Scl/Tal-1 is essential for development of all hematopoietic lineages. Cell, 86(1):47–57.
13. Real, P. J., Ligero, G., Ayllon, V., Ramos-Mejia, V., Bueno, C., Gutierrez-Aranda, I., Navarro-Montero, O., Lako, M., and Menendez, P. (2012). Scl/Tal1 regulates hematopoietic specification from human embryonic stem cells. Mol Ther, 20(7):1443–1453.
14. Shivdasani, R. A., Mayer, E. L., and Orkin, S. H. (1995). Absence of blood formation in mice lacking the T-cell leukaemia oncoprotein Tal1/Scl. Nature, 373(6513):432–434.
15. Soler, E., Andrieu-Soler, C., de Boer, E., Bryne, J. C., Thongjuea, S., Stadhouders, R., Palstra1, R.-J., Stevens, M., Kockx, C., van IJcken, W., Hou, J., Steinhoff, C., Rijkers, E., Lenhard, B., and Grosveld, F. (2010). The genome-wide dynamics of the binding of Ldb1 complexes during erythroid differentiation. Genes Dev, 24(3):277–289.
16. Stier, S., Cheng, T., Dombkowski, D., Carlesso, N., and Scadden, D. T. (2002). Notch1 activation increases hematopoietic stem cell self-renewal in vivo and favors lymphoid over myeloid lineage outcome. Blood, 99(7):2369–2378.
17. van der Meer, L. T., Jansen, J. H., and van der Reijden, B. A. (2010). Gfi1 and gfi1b: Key regulators of hematopoiesis. Leukemia, 24(11):1834–1843.
18. Visvader, J. E., Mao, X., Fujiwara, Y., Hahm, K., and Orkin, S. H. (1997). The lim-domain binding protein Ldb1 and its partner Lmo2 act as negative regulators of erythroid differentiation. PNAS, 94(25):13707–13712.
19. Zhang, Y., Payne, K. J., Zhu, Y., Price, M. A., Parrish, Y. K., Zielinska, E., Barsky, L. W., and Crooks, G. M. (2005). Scl expression at critical points in human hematopoietic lineage commitment. Stem Cells, 23(6):852–860.
